# Phylogeography of red muntjacs reveals three distinct mitochondrial lineages

**DOI:** 10.1186/s12862-017-0888-0

**Published:** 2017-01-26

**Authors:** Renata F. Martins, Jörns Fickel, Minh Le, Thanh van Nguyen, Ha M. Nguyen, Robert Timmins, Han Ming Gan, Jeffrine J. Rovie-Ryan, Dorina Lenz, Daniel W. Förster, Andreas Wilting

**Affiliations:** 1 Leibniz Institute for Zoo and Wildlife Research (IZW), Alfred-Kowalke-Straße 17, 10315 Berlin, Germany; 2 Potsdam University, Institute for Biochemistry and Biology, Karl-Liebknecht-Str. 22-24, 14476 Potsdam-Golm, Germany; 3 Faculty of Environmental Science, Hanoi University of Science, Vietnam National University, 334 Nguyen Trai Road, Hanoi, Vietnam; 4 Centre for Natural Resources and Environmental Studies, Vietnam National University, 19 Le Thanh Tong Street, Hanoi, Vietnam; 5 1123 Monroe Street, Evanston, IL 60202 USA; 6 School of Science, Monash University Malaysia, 47500 Bandar Sunway, Malaysia; 7 Genomics Facility, Tropical Medicine and Biology Multidisciplinary Platform, Monash University Malaysia, 47500 Bandar Sunway, Malaysia; 8 Department of Wildlife and National Parks (DWNP) Peninsular Malaysia, National Wildlife Forensic Laboratory (NWFL), 56100 Kuala Lumpur, Malaysia; 9 Present address: U.S. Agency for International Development, Governance for Inclusive Growth Program, Chemonics International Inc, 115 Tran Hung Dao Street, Hanoi, Vietnam

**Keywords:** Phylogeography, Archival DNA, Muntjac, Southeast Asia, Species complex

## Abstract

**Background:**

The members of the genus *Muntiacus* are of particular interest to evolutionary biologists due to their extreme chromosomal rearrangements and the ongoing discussions about the number of living species. Red muntjacs have the largest distribution of all muntjacs and were formerly considered as one species. Karyotype differences led to the provisional split between the Southern Red Muntjac (*Muntiacus muntjak*) and the Northern Red Muntjac (*M. vaginalis*), but uncertainties remain as, so far, no phylogenetic study has been conducted. Here, we analysed whole mitochondrial genomes of 59 archival and 16 contemporaneous samples to resolve uncertainties about their taxonomy and used red muntjacs as model for understanding the evolutionary history of other species in Southeast Asia.

**Results:**

We found three distinct matrilineal groups of red muntjacs: Sri Lankan red muntjacs (including the Western Ghats) diverged first from other muntjacs about 1.5 Mya; later northern red muntjacs (including North India and Indochina) and southern red muntjacs (Sundaland) split around 1.12 Mya. The diversification of red muntjacs into these three main lineages was likely promoted by two Pleistocene barriers: one through the Indian subcontinent and one separating the Indochinese and Sundaic red muntjacs. Interestingly, we found a high level of gene flow within the populations of northern and southern red muntjacs, indicating gene flow between populations in Indochina and dispersal of red muntjacs over the exposed Sunda Shelf during the Last Glacial Maximum.

**Conclusions:**

Our results provide new insights into the evolution of species in South and Southeast Asia as we found clear genetic differentiation in a widespread and generalist species, corresponding to two known biogeographical barriers: The Isthmus of Kra and the central Indian dry zone. In addition, our molecular data support either the delineation of three monotypic species or three subspecies, but more importantly these data highlight the conservation importance of the Sri Lankan/South Indian red muntjac.

**Electronic supplementary material:**

The online version of this article (doi:10.1186/s12862-017-0888-0) contains supplementary material, which is available to authorized users.

## Background

The number of recognized deer species has increased in recent decades, and it continues to do so due to rare discoveries of new forms in the wild, increased molecular efforts and the careful reexamination of museum collections. For example, the genus Muntiacus has increased in the number of named species through discovery of the Gongshan muntjac (*Muntiacus gongshanensis*) from the wild in 1990 [1], the Putao muntjac (*Muntiacus putaoensis*) from Myanmar, described based on molecular comparisons [2] and the Bornean Yellow muntjac (*Muntiacus atherodes*) described from museum skulls and skins in 1982 following a reappraisal of the muntjac taxa described previously from Borneo [3].

The genus *Muntiacus* Rafinesque (1815) comprises an undefined number of species and subspecies all native to South, Southeast and East Asia. Although muntjacs are studied for their dramatic variation in chromosome numbers [4, 5], taxonomic concordance within this genus has not been achieved yet, due to lack of molecular studies combined with, in some cases, limited morphological or ecological differences. Although the genus is mainly composed of endemics and species with small regional distributions (independent of the taxonomic revision), there are two exceptions: Reeve’s muntjac *M. reevesi*, which has a large native range largely confined to mainland China and even more striking, the red muntjacs. Even if different species/subspecies are considered, red muntjacs have the largest distributions of all muntjacs, ranging from the Indian subcontinent (Pakistan, India, Sri Lanka, Bangladesh, Nepal and Bhutan) to the Indochinese Peninsula (Vietnam, Laos, Myanmar, Thailand) and southeastern China and across the Malay Peninsula towards all the major islands of Sundaland (Borneo, Java, Sumatra) and Lesser Sunda Islands (Belitung, Bangka, Bali, Lombok, Lampung). In contrast to the majority of other muntjac species, red muntjacs occur in a wide range of habitats [6, 7]. They are generally forest-dwelling and occupy habitats from deciduous to evergreen forests and also occur in secondary forests and exotic commercial plantations [8, 9]. They have also been found in grasslands or shrubland savannahs, croplands and in altitudes commonly up to 1500 m asl (in extreme cases even up to 3500 m, [10]). Additionally, although habitat loss and hunting has already caused significant population declines throughout their range, in contrast to other ungulates in South and Southeast Asia, red muntjacs seem to be more resilient to habitat modifications and hunting [7].

Through most of the 20th century red muntjacs were generally classified as a single species *Muntiacus muntjak*, although it was noted by some authors that some forms might be better treated as distinct species (e.g. [11]). Groves in 2003 [12] elevated the mainland form to species level, designating it as *M. vaginalis*. As this study was solely based on a comparison between the karyotypes of one individual from Peninsular Malaysia and the karyotypes of Indochinese individuals, this classification has not been universally adopted. In 2008 *The IUCN/SSC Red List of Threatened Species* provisionally accepted this classification, but noted the need for corroborating studies. More recently, however, Groves and Grubb [13] described six ‘good’ species within the ‘red muntjac group’, based mainly on a few morphological characters and geographical distribution of populations (see Additional file 1: Table S1).

Here, we investigate the geographic distribution of mitochondrial lineages among red muntjac populations in order to address some of these taxonomic uncertainties, and discuss the results within the context of geological events that took place in Southeast Asia since the Pleistocene. This region’s geography has been heavily impacted by the climatic fluctuations of the Pleistocene, where the low sea levels during the glacial periods repeatedly exposed the shallow continental shelf, creating land corridors between the islands and the mainland [14]. While phylogeographic patterns of some species can be explained by the presence of these land corridors, those of others cannot; they seem to be the result of other vicariant events that took place in Southeast Asia [15, 16]. Characterising phylogeographic patterns within this widely distributed species complex will allow a better understanding of how climatic variations affected generalist species and will therefore help to understand the mechanisms leading to the evolution and speciation of mammals in the biodiversity hotspot of South and Southeast Asia.

## Methods

### Samples and DNA extraction

**Fig. 1:**
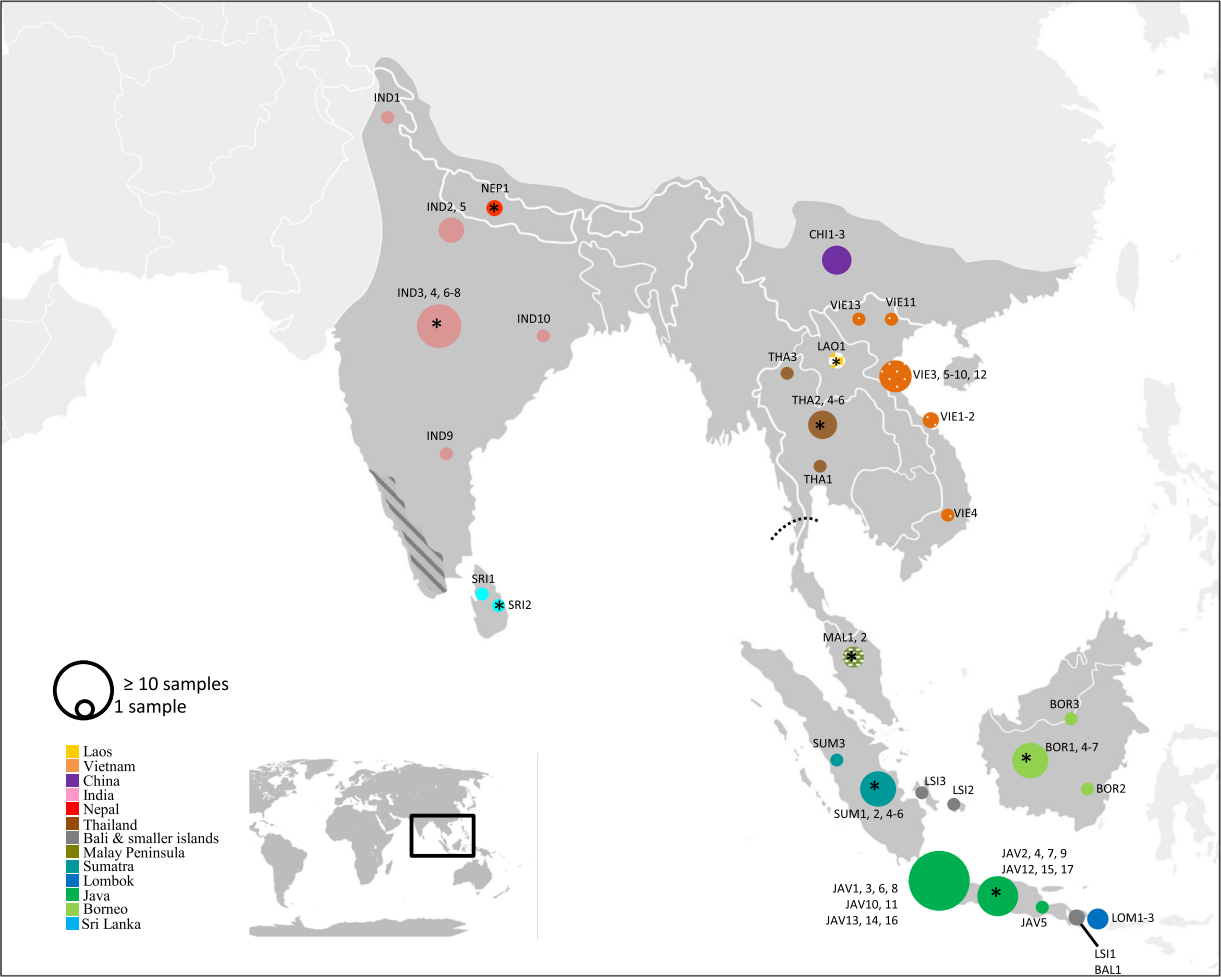
Illustrative map depicting the combined range of all red muntjacs (*dark grey*). The *dashed line* indicates the relative position of the Isthmus of Kra and the area indicated by *dark grey lines* indicates the relative position of the Western Ghats. *Circles* indicate sample origin according to colour and size is relative to sample size. *Checkered patterns* indicate contemporaneous samples, *while solid colours* indicate position of museum samples. * indicate samples for which only country of origin is available (See full samples details in Additional file 1: Table S1)

Archival samples from 92 red muntjacs were collected at several natural history museums (See final dataset in Additional file 1: Table S2). We collected nasal bones and dry tissue from skulls and skeletons, or drilled antlers when only this material was available. Only samples with a known locality were collected and archival samples from zoos were only included if they had a known wild origin. In addition, we also opportunistically collected 24 contemporary samples from Vietnam, Laos and Peninsular Malaysia (Fig. 1). Contemporaneous samples were all collected from dead animals during routine field surveys or confiscated trophies from hunters. All molecular work with archival samples was performed in a separate facility, operated under controlled conditions in order to minimize contamination. For DNA extraction of archival samples, we followed the DNeasy Blood & Tissue kit (Qiagen, Hilden, Germany) protocol with some modifications: digestion with Proteinase K occurred overnight at 56 °C and elution was preceded by an incubation period of 20 min. at 37^0^ C. DNA from fresh samples was extracted with the DNeasy Blood & Tissue kit (Qiagen) and the two samples from Peninsular Malaysia were extracted using conventional SDS/Proteinase K digestion followed by chloroform extraction [17]. Sham extractions were performed for archival and fresh samples and served as negative controls. They were processed like actual samples with every step and included in follow-up PCR reactions.

### Library building and hybridization capture

All samples, including extraction negative controls, were processed into libraries for sequencing with either the Ion Torrent Personal Genome Machine (PGM; Thermo Fischer Scientific, USA) or the MiSeq (Illumina, San Diego, CA, USA). For PGM libraries we followed the manufacturer’s protocol with modifications: we used the gDNA plus Fragment Library Kit (Thermo Fisher Scientific, USA) but all reactions were performed in a quarter of the suggested volume and blunt ending included a heat inactivation of the enzyme (20 min at 72 °C), so that no intermediate purification step was necessary. Double-stranded sequencing libraries were also prepared for the Illumina MiSeq platform according to the protocol of Fortes and Paijmans [18] with single 8 nt indexing. For two samples from Peninsular Malaysia, purified gDNA was sheared to 500 bp using Covaris instrument (Woburn, MA) and subsequently prepared using NEB Next Ultra DNA library prep kit for Illumina (New England Biolabs, Ipswich, MA) with dual 8 nt indexing. The constructed libraries were sequenced on the MiSeq system located at the Monash University Malaysia Genomics Facility with the run configuration of 2 x 250 bp.

Because DNA extracted from museum samples is expected to be highly degraded and because external contamination from handling, storage and exposure may have introduced foreign DNA, we performed a hybridization capture step prior to sequencing in order to enrich the archival samples for their mitochondrial DNA (mtDNA). To capture target mtDNA, we created baits from a fresh sample of the closely related species *Muntiacus reevesi* (blood sample from Zoo Münster, Germany). The baits were generated by amplifying the whole mitochondrial DNA via long range PCR (primer sequences and PCR conditions described in Additional file 1: Table S3). Sheared and pooled long range PCR fragments were then prepared as bait [19]. Hybridization capture also followed the protocol described in [19]. For PGM sequencing the capture mixture was modified and included the blocking oligos for the PGM adapters.

Libraries from fresh samples and captured archival DNA libraries were pooled equimolarly, but never together in the same run, and sequenced with either the Ion PGM™ Sequencing 200 Kit v2 or the Illumina MiSeq kit v3 (150-cycle), following the respective manufacturers’ protocols.

### Bioinformatic analyses

Sequence reads from Illumina were de-multiplexed according to the respective indexes with the Illumina software bcl2fastq v2.17 (Illumina, San Diego, CA, USA) and adapters were clipped from the sequence reads with the software cutadapt v1.3 [20]. Sequencing reads generated with the Ion Torrent PGM were first processed with the inbuilt software (de-multiplexing and adapter clipping). Quality trimming was done through a sliding window approach (10 bp; Q20) and quality filtering then proceeded by removing all reads shorter than 20 bp from the analyses. The complete mitochondrial genome sequence of *M. muntjak* (NCBI Accession Nr. NC_004563.1) was used as reference for mapping of the sequencing reads, which was done with the software BWA v.0.7.10 [21]. A following quality filtering step was performed to remove duplicate reads from the dataset, so that only unique reads were kept, with the software MarkDuplicates from picard-tools v.1.106 (http://picard.sourceforge.net/). Indels were called with bcftools v.1.2 (http://github.com/samtools/bcftools) and variant calling was carried out in GATK v.1.6 [22], with N-masking of positions with less than 3 unique reads and ambiguous heterozygous positions. Variants were called based on the majority rule. Only sequences with 85% or more of the genome covered with 3x or greater depth were accepted for analysis (range of depth was between 6.87 and 294.49x, with a mean of 72.35x, see sequencing results in Additional file 1: Table S4) and missing data was included for analyses. Finally, potential presence of *numts* was investigated by searching for the presence of stop codons in coding genes and indels; and we found no evidence of *numts* in our dataset. All mitogenomes obtained were deposited in Genbank (KY052082-KY052156) and accession numbers for each sample are given in Additional file 1: Table S1.

### Genetic diversity, divergence dating and population demography

All *Muntiacus* sequences obtained in this study were aligned using mafft v.7.245 [23] with the auto setting..We constructed a median joining (MJ) network with network v.4.6.1.4 [24], with missing/gaps sites and invariant sites removed from the dataset. Haplotype diversity and nucleotide diversity were calculated in DnaSP v.5.10 [25]. Analysis of molecular variance (amova) and population differentiation (F_st_) were calculated with Arlequin v.3.5.1.2 [26]. Samples were grouped according to geographical origin into populations and populations belonging to different clades were considered groups, so that we had three different groups: Mainland, Sunda and Sri Lanka.

To estimate divergence times of different muntjac clades and population demographic changes over time, we inferred genealogies using a relaxed lognormal clock model as implemented in BEAST v.1.8.1 [27]. We performed this analysis by creating a dataset with ten other Cervidae species (at least one representative species from each tribe from the three subfamilies of Cervidae) and one Bovidae species (*Bos javanicus*) in order to estimate the divergence time and mutation rate for the muntjac clade. The root age (the most recent common ancestor [TMRCA] of Bovids and Cervids) was set to 16.6 million years (My) [28], with a normal prior distribution and standard deviation of 2. This date represents the minimum age of fossil evidence between Cervidae and Bovidae [29]. Another study reported similar divergence times [27] and dated the split between Reeve’s muntjacs (*M. reevesi*) and red muntjac + black muntjac (*M. crinifrons*) clade at 3 Mya [30]. We subsequently used the estimated red muntjac split as tree prior for the calibrations within our dataset.

Using the complete mtDNA we employed RAxML GUI v.1.5 [31] to reconstruct a maximum likelihood (ML) phylogenetic tree of red muntjacs applying the substitution model determined by jModelTest v.2.1.7 (GTR + G + I, [32]). Both the ML tree and the MJ network revealed the presence of three monophyletic clades comprising individuals from three geographically distinct regions. Based on these results we created three different data sets and analyzed them independently with BEAST v.1.8: 1) full data set comprising all individuals from the three identified clades; 2) ‘Mainland’ clade only; and 3) ‘Sunda’ clade only. As analyses 2 and 3 describe distinct population groups, they are more suited for demographic analyses that assume a coalescent process. We did not generate a data set comprising only Sri Lankan samples (which constituted the third clade) due to the few individuals sampled. For analyses of the full dataset (dataset 1) we inferred the phylogeny with BEAST v1.8 using a lognormal clock model and a Yule speciation tree model (assuming a constant lineage birth rate for each branch), with GTR + G + I as substitution model determined by jModelTest (based on AIC). We set the root height to 1.5 Mya as inferred by the Cervidae/Bovidae phylogenetic tree and estimated the clock rate. For the demographic analysis (datasets 2 and 3) we assumed the same substitution model and the coalescent prior of Extended Bayesian Skyline as tree prior as implemented in BEAST v1.8. To test the hypothesis of a recent expansion of red muntjac populations we set the root height at 1.12 Mya, as inferred from the divergence dating phylogenetic tree. For every analysis we performed two independent runs with 50 million iterations each, sampling one tree per 5000 iterations. Results of each run were visualized with tracer v.1.6 (implemented in Beast v1.8). The first 10% per run (1000 trees) was discarded as burn-in and the remaining trees were combined with logcombiner v.1.8.1 (implemented in Beast v.1.8). Maximum credibility trees were obtained with treeannotator, also distributed as part of the BEAST package. Skyline plots were generated using the R package ggplot2 [33].

As we did not obtain any samples from the Western Ghats, in Southern India, we included five cytochrome *b* (cyt*b*) sequences from NCBI (Accession numbers EU727189; FJ190162; FJ190160; JN861030; JN861033) to test their phylogenetic placement. We aligned these sequences with cyt*b* sequences extracted from the complete mitogenome dataset using MAFFT v.7.245 with specifications as before. RAxML GUI v.1.5 was used to construct the ML tree. We visualized and edited all trees with FigTree v.1.4.2 (http://tree.bio.ed.ac.uk/software/figtree/).

## Results

After quality filtering and mapping of all reads, we were able to obtain a final dataset of 16386 bp of the complete mitogenome for a total of 59 archival and 16 contemporary samples, which constituted a total of 65 different haplotypes. Number of variable and parsimony informative sites was high for all coding genes (Additonal file 1: Table S5).

### Phylogeography and population genetic diversity

**Fig. 2:**
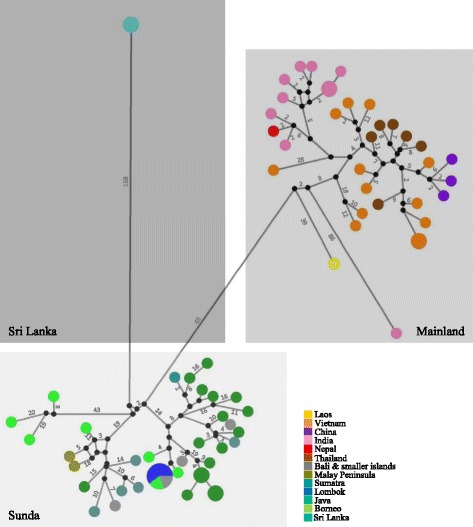
Median joining network of full mitogenome of all archival and contemporaneous red muntjac samples. *Circle size* is proportional to haplotype frequencies; *fill color* denotes geographical origin; *lines* represent one mutational step, except when indicated otherwise with numbers. *Black circles* represent missing vectors. The three major clades are denoted in the different boxes and indicated by name

**Fig. 3:**
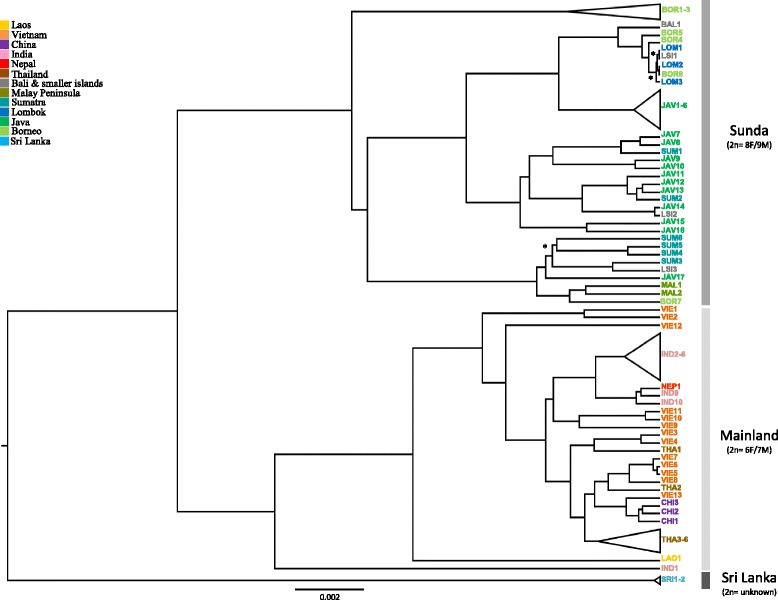
Maximum credibility tree based on 59 archival and 16 contemporaneous *red muntjac* samples, spanning across *red muntjacs* combined distribution. All branches are supported with BPP > 0.9 except the ones marked with * where BPP > 0.8. This phylogenetic relationship was reconstructed with BEAST and the root age was constrained to 3 Mya as suggested by the divergence dating analyses. Clades of similar sequences have been collapsed and the detailed information about all individuals can be found in Table S1

All three analytical approaches, MJ network (Fig. 2), Bayesian inference (Fig. 3) and maximum likelihood analysis (Additional file 1: Figure S1) revealed three well supported mtDNA clades Sequences from two major clades originated from (i) mainland South and Southeast Asia and China (henceforth referred to as Mainland) (ii) Peninsular Malaysia and the Sunda Islands (Sunda); while the third clade consisted exclusively of sequences originating from Sri Lanka (Sri Lanka). Haplotype and nucleotide diversity for each clade was similar (Table 1). F_ST_ values indicated substantial genetic structuring among the three clades: the highest differentiation was found between Mainland and Sri Lanka (0.906, *p* <0.05), the second highest differentiation was between Sunda and Sri Lanka (0.848, *p* <0.001) and the lowest, yet still very high, between Mainland and Sunda (0.631, *p* <0.001).

**Table 1:** Summary of number of samples and haplotypes distributed in each clade and measurements of diversity indexes of the three major red muntjac clades. Haplotypic diversity (h) and nucleotide diversity (π) could not be measured for Sri Lanka clade due to the low sample number

	Mainland	Sunda	Sri Lanka
N	33	40	2
Haplotypes	31	33	1
Haplotype diversity (h)	0.996	0.982	―
Nucleotide diversity (π)	0.006	0.009	―

The Mainland clade comprised all samples from mainland South and Southeast Asia and China, excluding samples from Sri Lanka and Peninsular Malaysia. In general, samples with the same geographic origin formed cohesive branches, except individuals from Vietnam and Thailand which were placed sporadically together or clustered with the Chinese samples. Interestingly, one single individual sampled from Himachal Pradesh province in India (IND1) and one individual from Laos (LAO1) formed two basal branches in this clade (Fig. 3). Conversely, the Sunda clade comprised all red muntjacs from the Sunda Shelf, including individuals from Peninsular Malaysia, which were closely related to an individual from Borneo with no further indication of phylogeographic sub-structuring of samples from different land-masses in the Sunda Shelf. Amova results (Table 2) corroborate these findings, showing that variation is highest among the three groups tested (60.5%, p-value <0.001) and lowest among populations within groups (10.6%, p-value <0.001). The ML inference based on the cyt*b* gene only (Additional file 1: Figure S2), which included Western Ghats red muntjac samples, revealed a sister relationship between Western Ghats and Sri Lankan red muntjacs.

**Table 2:** Amova results among groups (Mainland, Sunda, Sri Lanka), among populations within groups (populations were defined based on country of origin) and within populations. Results show the majority of variation explained among groups, indicating differentiation between the three major clades

	d. f.	Variance components	Percentage of variation^a^
Among groups	2	94.1	60.5
Among population within groups	8	16.5	10.6
Within populations	64	45	28.9

^a^all *p*-values <0.001

### Divergence times and demographic changes

**Fig. 4:**
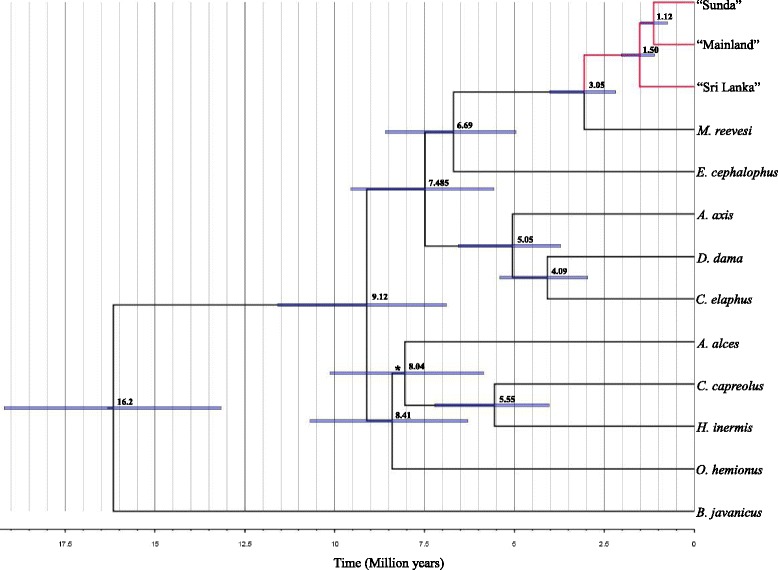
Divergence dating maximum credibility tree obtain with BEAST. Root age was calibrated to 16.6 Myr and indicates split age between Bovidae and Cervidae (16.2 Mya) and between 10 different other Cervidae species. Branches highlighted in *red* indicate the clades analysed in this study

Based on the calibrated root age (TMRCA of Bovids and Cervids at 16.6 ± 2 Mya) our results suggested that the split between the red muntjacs and other *Muntiacus* species occurred in the late Pliocene around 3 Myr (95% High posterior density [HPD] = 1.7 – 5.7) (Fig. 4). Within red muntjacs, the split between the Sri Lankan matriline and the other red muntjacs was estimated to have occurred at around 1.5 Mya (95% HPD = 0.78 – 2.82), while the divergence time between the two other major clades was inferred to be around 1.12 Mya (95% HPD = 0.5 – 2.22). Divergence dates obtained were associated with relatively small 95% posterior density intervals.

**Fig. 5:**
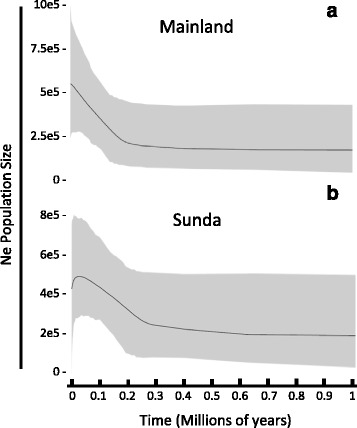
Extended Bayesian Skyline Plots of female effective population size changes of two major clades (**a**) Mainland and (**b**) Sunda within *red muntjacs* through time. *Black line* represents the mean number of Ne changes through time and *grey area* denotes the 95% High Probability Density

The inferences of effective population sizes showed different demographic histories for each of the two clades analysed (Fig. 5). Although both experienced a population expansion in the late Pleistocene (Mainland clade about 0.2 Mya, Sunda clade about 0.27 Mya), the increase in the mainland was maintained while the effective population size of Sundaic red muntjacs started to steeply decline about 25 thousand years ago (Fig. 5).

## Discussion

### Phylogeographic patterns and population structure

Investigating variation in complete mitochondrial genomes across the red muntjac distribution range enabled us to examine the evolutionary history of these populations, including phylogeographic structure and historical demography. The high genetic diversity within, and large divergence among the main clades indicate a long-term separation of these clades, which in turn implies that red muntjacs had spread across South and Southeast Asia shortly after their split from other muntjac species. We found, however, evidence for two geographic barriers that (i) separated populations from northern India and Sri Lanka + Western Ghats and (ii) separated Sundaic and Mainland populations.

The Sri Lanka clade split first from the other two populations around 1.5 Mya and our data provides the first molecular evidence for its distinction. Due to the low number of samples obtained from this region we were unable to further assess their evolutionary history and genetic diversity. Other authors, who regarded the population of Sri Lanka as a separate taxonomic unit (either as a full species *M. malabaricus* – [13] or as a subspecies *M. m. malabaricus* – [10]) have grouped the populations from the Indian Western Ghats together with the Sri Lankan red muntjacs. The results from the ML tree based on the cyt*b* gene support this inclusion and provide another example of the distinct populations in the ‘Western Ghats - Sri Lanka biodiversity hotspot’ [34]. Examples of a discontinuity in species distribution between Northern and Southern India are numerous and this disjunct distribution seems particularly pronounced in wet-zone species [35]. The dry zone of central India (with rainfall of just 50 – 100 cm per year) seems to be an unsuitable habitat for many species and has often led to speciation between populations isolated in more suitable forested habitats (refugia) in the northern and southern regions of the Indian subcontinent (e.g., the Asian fairy-bluebird *Irena puella* [36]; Flying lizards *Draco* genus [37]; Nilgiri tahr *Nilgiri tragushylocrius* [38]). Red muntjacs, however, are much more versatile and currently occur to the authors’ knowledge generally throughout the ‘dry zone’. This implies that the barrier to gene flow has existed in the past, presumably as a result of extreme dry climatic conditions caused by global ice advances. For red muntjacs, the isolation of populations in the southern wet-zones (Sri Lanka and Western Ghats) might have persisted even after the re-colonization of the dry zone of central India, if in fact other barriers to gene flow, such as karyotype differences, had arisen in the interim, although at present no such evidence exists (see below).

The second major clade was composed of all Indian and Indochinese samples. The phylogenetic mtDNA tree showed geographical structuring and the basal placement of two individuals that came from Laos and from Himachal Pradesh Province in India. Their position (and the relatively long branch distance in the network analysis) suggests ancient isolation of localized populations during the Pleistocene. It is likely that populations in Indochina and India were repeatedly affected by climatic fluctuations of the Pleistocene, resulting in some areas of their current continuous range having become unsuitable (most probably too dry and supporting only very open habitat). Currently and presumably also during previous interglacials, when forest conditions predominated, red muntjacs would have expanded their range and reoccupied former distribution areas. An example of such phylogeographic pattern of a “colonization from the east” was observed in leopard cats (*Prionailurus bengalensis*), where populations became isolated in their refugia due to the drier and unsuitable habitat in the rest of the Indian subcontinent [39]. This effective population size expansion is reflected in the Bayesian skyline plot and is likely responsible for the patterns of admixture we observed in our ML and BI trees, where samples from Vietnam, Thailand, China and India are closely related in the most derived branches of the trees. We estimated that this expansion started around 200 kya, a time marked by the beginning of an interglacial period (240 – 190 kya). This period succeeded a glacial time, with sea levels as low as −130 m below present [40] that would have provoke drier climate in continental areas.

The surprising placement of the Himachal Pradesh Province (IND1) sample at a basal position allows us to speculate as well that there may exist a distinct ‘high elevation Himalayan red muntjac’ (also supported by RJT’s unpublished morphological examinations), which may have evolved in refugial areas during these dry periods. Unfortunately, we could not include samples from Hainan Island, from high elevation Himalayan populations, from the Cardamom Mountains of Thailand and Cambodia, from the Southern Annamites of Vietnam, from Northern Myanmar, or from the Indian dry zones in our analyses (either samples had no proper location or yielded insufficient data); samples that from a phylogeographic perspective might provide further resolution or additional lineages. These regions correspond in part to the distribution of two currently described muntjac taxa: the sub-/species *M. (m.) nigripes*, described from Hainan Island and considered by some authors to occur also in northernmost Vietnam and Yunnan (China) and the sub-/species *M. (m.) aureus*, described to occur in northwestern India and considered to also occur in central India and disjunctly in Myanmar [13]. Thus, further genetic substructuring may exist within the Mainland clade, which could be unveiled with more intensive and extensive sampling.

The third clade, the Sunda clade, included all samples from Sundaland (including the lower Malay Peninsula). The very clear distinction of this clade indicated the existence of a long lasting migration barrier preventing gene flow between populations possibly north and south of the Isthmus of Kra, a recognized phyto- and zoogeographic boundary located on the Malay/Thai Peninsula around 10°30’ N. Although the Isthmus of Kra separates the Indochinese and Sundaic subregions [41], studies on different taxa revealed that the Isthmus of Kra is not a clear geophysical barrier, but rather a transition zone ranging from 5° N to 13° N (e.g. bats and birds [42]; butterflies [43]; amphibians [44]; mammals [45]). As we had only two samples from Peninsular Malaysia, we could not address the exact latitude of this separation (or indeed whether there is in fact introgression as suggested by morphological samples – RJT unpublished data). Increased sampling efforts would therefore be required to identify the true nature and geographic location of the ‘boundary’ between northern and southern red muntjac populations. Nuclear DNA analyses are also needed to address questions of a presumed secondary contact zone and potential hybridization of northern and southern red muntjacs on the Malay Peninsula. Interestingly, a recent multi-species study on mammals showed that no clear physical barrier is needed to maintain the separation of the Sundaic and Indochinese faunas, but that instead a combination of different climatic conditions during the Pleistocene and species-specific life history traits are sufficient to result in the observed pattern (Radchuk, unpublished data). With respect to the red muntjacs, these findings could indicate that northern and southern red muntjacs evolved different ecological adaptations during the periods their ranges became restricted, which is indicated by the subsequent population expansions in the EBSPs of both clades.

Within the Sunda clade we found evidence of the effects of Toba super volcanic eruption in Northern Sumatra around 74 thousand years ago. The Toba super eruption is described as one of the greatest eruptions in the last two million years [46]. It created an ash cloud that would have covered the northern part of Sumatra and south of Peninsular Malaysia leading to changes in vegetation and possible local extinctions of mammal species [47–49]. Our results support such potential extinctions of red muntjacs in Peninsular Malaysia and Sumatra, as the analysed individuals all derived from Bornean or Java populations, which occupy all basal positions of the internal nodes. This pattern suggests that Sumatra and Peninsular Malaysia were colonized, more than once, from Bornean and Javan populations, potentially after the Toba eruption. Such a re-colonisation of the southern Malay Peninsula would have been facilitated by the existing land bridges throughout the Late Pleistocene between the larger Sunda Islands. These land bridges also explain the lack of geographical sub-structuring as seen both in the MJ network and in the gene trees within the Sunda clade, as the exposed shelf allowed gene flow among populations on all larger landmasses. Being habitat generalists red muntjacs could have easily colonized new habitats on the exposed shelf. After the Last Glacial Maximum, rising sea levels not only separated the larger landmasses but also drastically reduced the available land area in the Sunda Shelf. This reduction in land and thus habitat availability coincides with the observed decrease in population size observed in the skyline inferences. However inferred divergence times should still be considered rough estimates, because they depend on estimated mutation rates.

### Taxonomic implications

Currently, up to six species of red muntjacs have been described. The most commonly accepted split within the red muntjacs is the one separating the mainland and Sundaic forms in two species: Northern red muntjac *Muntiacus vaginalis* and Southern red muntjac *M. muntjak*. Because species delineation was based only on the karyotype of one individual from Peninsular Malaysia and on a few morphological traits, this classification is still applied with some reservations. The proposed additional division of mainland (Northern) red muntjacs into different species [13, 50] is likewise weak and seldom adopted. In that study [13], differences were mainly described based on morphological traits, but the described variations are more likely trait polymorphisms within a largely distributed species, rather than distinctive morphological characters [7]. The molecular data presented here does not support the delineation of six red muntjac species as we did not find six distinct matrilines. Instead, we found evidence for a deep split between Sundaic (southern) and mainland (northern) red muntjacs, which would be concordant with karyotypic evidence: the few examined individuals of Sundaic forms had 2n = 8 F/9 M, while the most frequently studied Mainland forms had 2n = 6 F/7 M [13]. The evidence presented here clearly shows the existence of a third distinct Sri Lankan + Western Ghats clade, for which the karyotype characterization is still lacking. Our molecular data supports likewise the recognition of the Sri Lankan population as a distinct taxonomic unit, as this lineage split first from all other red muntjacs at around 1.5 Mya. So far the Sri Lankan and Western Ghats populations have been recognized as *M. (m.) malabaricus* due to their smaller body size (compared to more northerly and eastern mainland red muntjacs) and due to some pelage coloration differences [13].

The three clades introduced here are distinguished by deep molecular splits and appear to be geographically separated. We believe that the results of this study provide a good basis for a future taxonomic reassessment. We refrain from assigning these clades species or subspecies rank because: a) the estimated divergence dates and the observed pairwise differences are in the range of both recognized species [5] and recognized subspecies [51] and thus are not decisive, b) we only analysed mtDNA and thus have no information on potential incomplete lineage sorting, and c) our analyses lacked samples from regions where the three clades must meet and potentially overlap (within central and southern India and probably within the transition zone of the Isthmus of Kra). Therefore, further sampling and analysis of nuclear DNA data, and potentially also of morphological and karyotype data is needed, since both characteristics are further indicators of barriers to gene flow.

Independent of their specific taxonomic assignment as species or subspecies, our data clearly advocate the distinct management of the populations in these clades. Despite the indiscriminate hunting pressure on larger mammals in Southeast Asia, particularly in Indochina [52], both Northern and Southern red muntjacs are still widespread and less threatened than other species in this region. Our data did not uncover any populations of greater conservation concern among Mainland or Sundaic red muntjacs, although more extensive sampling might reveal the existence of taxonomic units of conservation concern. However, our data do clearly reveal the distinctiveness of the Sri Lankan + Western Ghats populations. The spatial restriction of this clade and ongoing threats both in Sri Lanka and also the Western Ghats [53] highlight the conservation significance of red muntjacs in this region. Additional field surveys, including further molecular sampling, are important to assess and monitor the conservation status of *M.* (*m.*) *malabaricus*.

## Conclusions

We found substantial genetic differentiation in a widespread species, corresponding to at least two biogeographical barriers located in the major biodiversity hotspot of South and Southeast Asia: the Isthmus of Kra in northern Peninsula Malaysia and the central Indian dry zone. However, within each of the three lineages we found evidence of mitochondrial admixture between populations which are now geographically isolated, suggesting that red muntjacs, as a generalist species, could utilize land corridors exposed during the low sea level periods of the Pleistocene. Our results, finally, indicate for the first time molecular evidence for the distinctiveness of the Sri Lanka and Western Ghats red muntjac populations.

## Additional file

Additional file 1:
**Table S1.** Comparison of number of accepted red muntjac species and subspecies among three different recent publications. Number of recognized species range from 1 to 6 and number of recognized subspecies range from none to 10. **Table S2.** Complete dataset used for analyses, with information on origin, location and contact. **Table S3.** Long-range PCR primer sequences and annealing temperatures used for bait development. **Table S4.** Sequencing results from all samples used in this study, with indication of sequencing platform, percentage of reads on target, average base coverage depth and percentage of reference genome covered with at least 3x coverage. **Table S5.** Number of Variable sites (V) and Parsimony informative sites (Pi) per coding gene, throughout the full mitogenomes obtained. **Figure S1.** Best tree obtained with RAxML through Maximum Likelihood Analysis. **Figure S2.** Maximum Likelihood tree of the Cytochrome B gene, including five individuals from Western Ghats, supporting the clade differentiation of Sri Lanka – Western Ghats from all other red muntjac populations. (PDF 519 kb)

## Abbreviations

Asl: Above sea level
BPP: Bayesian posterior probability
gDNA: Genomic DNA
mtDNA: Mitochondrial DNA
Mya: Million years ago
