# Supplementary material for: Phylogeography of red muntjacs reveals three distinct mitochondrial lineages
Source: BMC Evol Biol. 2017 Jan 26;17:34. doi: 10.1186/s12862-017-0888-0 (PMC5267393; doi:10.1186/s12862-017-0888-0)
Supplement: Additional file 1: — Table S1. Comparison of number of accepted red muntjac species and subspecies among three different recent publications. Number of recognized species range from 1 to 6 and number of recognized subspecies range from none to 10. Table S2. Complete dataset used for analyses, with information on origin, location and contact. Table S3. Long-range PCR primer sequences and annealing temperatures used for bait development. Table S4. Sequencing results from all samples used in this study, with indication of sequencing platform, percentage of reads on target, average base coverage depth and percentage of reference genome covered with at least 3x coverage. Table S5. Number of Variable sites (V) and Parsimony informative sites (Pi) per coding gene, throughout the full mitogenomes obtained. Figure S1. Best tree obtained with RAxML through Maximum Likelihood Analysis. Figure S2. Maximum Likelihood tree of the Cytochrome B gene, including five individuals from Western Ghats, supporting the clade differentiation of Sri Lanka – Western Ghats from all other red muntjac populations. (PDF 519 kb) [file 12862_2017_888_MOESM1_ESM.pdf]

Phylogeography of red muntjacs reveals three distinct mitochondrial lineages

Renata F. Martins, Jörns Fickel, Minh Le, Thanh van Nguyen, Ha M. Nguyen, Robert Timmins, Han Ming Gan, Jeffrine J. Rovie-Ryan, Dorina Lenz, Daniel W. Förster, Andreas Wilting

Additional file 1

Table S1. Comparison of number of accepted red muntjac species and subspecies among three different recent publications. Number of recognized species range from 1 to 6 and number of recognized subspecies range from none to 10.

	Mattioli (2011)[10]	Grubb and Groves (2011)[13]	Timmins *et al*. (2016)[7]
Species	*M. muntjak*	*M. muntjak*<br>*M. vaginalis*<br>*M. malabaricus*<br>*M. aureus*<br>*M. nigripes*<br>*'M. guongdongensis'**	*M. muntjak*<br>*M. vaginalis*
Subspecies	*M. m. muntjak*<br>*M. m. annamensis*<br>*M. m. aureus*<br>*M. m. curvostylis*<br>*M. m. malabaricus*<br>*M. m. mengalis*<br>*M. m. montanus*<br>*M. m. nigripes*<br>*M. m. vaginalis*<br>*M. m. yunnanensis*	*M. v. vaginalis*<br>*M. v. curvostylis*	not applicable

* Identified as *Incertae sedis*, but referring to the different form described by Li J-x &Xu, 1996

Table S2. Complete dataset used for analyses, with information on origin, location and contact.

Collection ID	Sample name	Name in Figures	Genbank	Source material	Geographic origin	Geographic origin	Year	Contact
46238	MMU1	BAL1	KY052120	nasal bone	West Bali	West Bali		Dr. Frieder Mayer, Naturkundemuseum Berlin
46239	MMU2	JAV13	KY052121	nasal bone	West Java	Java		Dr. Frieder Mayer, Naturkundemuseum Berlin
17534	MMU3	THA2	KY052091	nasal bone	Thailand	Thailand		Dr. Frieder Mayer, Naturkundemuseum Berlin
91131	MMU6	IND5	KY052092	nasal bone and tissue from skull	North India	North India		Dr. Frieder Mayer, Naturkundemuseum Berlin
91143	MMU8	IND2	KY052093	nasal bone	North India	North India		Dr. Frieder Mayer, Naturkundemuseum Berlin
40556	MMU10	CHI3	KY052094	nasal bone	Yunnan China	China		Dr. Frieder Mayer, Naturkundemuseum Berlin
4837	MMU12	IND10	KY052095	nasal bone	East India	East India		Dr. Frieder Mayer, Naturkundemuseum Berlin
34074	MMU16	BOR2	KY052127	nasal bone	Southeast Borneo	Borneo		Dr. Frieder Mayer, Naturkundemuseum Berlin
92292	MMU17	LOM2	KY052122	bone from skull	Lombok	Lombok		Dr. Frieder Mayer, Naturkundemuseum Berlin
70927	MMU18	SRI1	KY052116	nasal bone	East Sri Lanka	Sri Lanka		Dr. Frieder Mayer, Naturkundemuseum Berlin
33459	MMU20	CHI1	KY052096	nasal bone	Yunnan China	China		Dr. Frieder Mayer, Naturkundemuseum Berlin
92296	MMU21	LSI1	KY052123	tissue from skull	Lesser Sunda Islands	Lesser Sunda Islands		Dr. Frieder Mayer, Naturkundemuseum Berlin
70928	MMU22	BOR7	KY052124	nasal bone	Borneo	Borneo		Dr. Frieder Mayer, Naturkundemuseum Berlin
ZMA 10490	MMU24	SUM6	KY052125	nasal bone	Sumatra	Sumatra	1950	Pepijn Kamminga, Naturalis Leiden
ZMA 10491	MMU25	SUM1	KY052126	nasal bone	Sumatra	Sumatra	1951	Pepijn Kamminga, Naturalis Leiden
16699	MMU27	JAV17	KY052128	nasal bone and tissue from skull	Java	Java	1856	Dr. Stefan Merker, Staatliches Museum für Naturkunde Stuttgart
16701	MMU28	JAV15	KY052129	nasal bone	Java	Java	1878	Dr. Stefan Merker, Staatliches Museum für Naturkunde Stuttgart
50998	MMU29	JAV5	KY052130	nasal bone	East Java	Java	1950	Dr. Stefan Merker, Staatliches Museum für Naturkunde Stuttgart
15974	MMU30	BOR5	KY052131	antler drill	Borneo	Borneo	1902	Dr. Stefan Merker, Staatliches Museum für Naturkunde Stuttgart
15968	MMU31	BOR6	KY052132	antler drill	Borneo	Borneo	1902	Dr. Stefan Merker, Staatliches Museum für Naturkunde Stuttgart

15979	MMU32	BOR4	KY052133	antler drill	Borneo	Borneo	1902	Dr. Stefan Merker, Staatliches Museum für Naturkunde Stuttgart
97.679	MMU34	THA6	KY052097	nasal bone	Thailand	Thailand	1900	Dr. Rainer Hutterer, Zoologisches Forschungsmuseum Alexander Koenig Bonn
2886	MMU35	JAV6	KY052134	nasal bone	West Java	Java	1930	Dr. Frank Zachos, Naturhistorisches Museum Wien
2887	MMU36	JAV11	KY052135	nasal bone	West Java	Java	1930	Dr. Frank Zachos, Naturhistorisches Museum Wien
1468	MMU38	SUM4	KY052136	nasal bone	Sumatra	Sumatra	1904	Dr. Frank Zachos, Naturhistorisches Museum Wien
2120	MMU40	SUM2	KY052137	nasal bone	Sumatra	Sumatra	1852	Dr. Frank Zachos, Naturhistorisches Museum Wien
3022	MMU41	JAV14	KY052138	nasal bone	West Java	Java	1858	Dr. Frank Zachos, Naturhistorisches Museum Wien
2931	MMU42	JAV1	KY052139	nasal bone	West Java	Java	1929	Dr. Frank Zachos, Naturhistorisches Museum Wien
2119	MMU43	JAV16	KY052140	nasal bone	West Java	Java	1858	Dr. Frank Zachos, Naturhistorisches Museum Wien
3178	MMU44	JAV3	KY052141	nasal bone	West Java	Java	1929	Dr. Frank Zachos, Naturhistorisches Museum Wien
2052	MMU46	JAV8	KY052142	nasal bone	West Java	Java	1858	Dr. Frank Zachos, Naturhistorisches Museum Wien
2117	MMU48	SUM5	KY052143	tissue	Sumatra	Sumatra	1886	Dr. Frank Zachos, Naturhistorisches Museum Wien
B3399	MMU49	JAV7	KY052144	skin	Java	Java	1919	Dr. Frank Zachos, Naturhistorisches Museum Wien
B3400	MMU50	JAV2	KY052145	skin	Java	Java		Dr. Frank Zachos, Naturhistorisches Museum Wien
B3402	MMU51	JAV4	KY052146	skin	Java	Java		Dr. Frank Zachos, Naturhistorisches Museum Wien
1497	MMU53	SUM3	KY052147	bone drill	Sumatra	Sumatra	1904	Dr. Frank Zachos, Naturhistorisches Museum Wien
35585	MMU59	LOM1	KY052148	tissue	Lombok	Lombok		Dr. Irina Ruf, Senckenberg Forschungsinstitut Mammalogie Frankfurt
34878	MMU60	LOM3	KY052149	tissue	Lombok	Lombok		Dr. Irina Ruf, Senckenberg Forschungsinstitut Mammalogie Frankfurt
15953	MMU62	IND6	KY052098	tissue	India	India		Dr. Irina Ruf, Senckenberg Forschungsinstitut Mammalogie Frankfurt
M122	MMU64	JAV9	KY052150	tissue from skeleton	Java	Java		Mogens Andersen, Natural History Museum of Denmark Copenhagen
M123	MMU65	NEP1	KY052099	nasal bone	Nepal	Nepal	1838	Mogens Andersen, Natural

								History Museum of Denmark Copenhagen
M505	MMU67	LSI2	KY052151	nasal bone	Belitung Island	Belitung Island	1887	Mogens Andersen, Natural History Museum of Denmark Copenhagen
M537	MMU68	IND7	KY052100	nasal bone and tissue from skull	India	India	1887	Mogens Andersen, Natural History Museum of Denmark Copenhagen
M1267	MMU72	THA4	KY052101	tissue from skull	Thailand	Thailand	1926	Mogens Andersen, Natural History Museum of Denmark Copenhagen
M125	MMU74	THA1	KY052102	Tissue in alcohol	South Thailand	Thailand	1877	Mogens Andersen, Natural History Museum of Denmark Copenhagen
46109	MMU75	IND9	KY052103	skin	South India	South India		Dr. Frieder Mayer, Naturkundemuseum Berlin
91138	MMU81	IND3	KY052104	skin	India	India		Dr. Frieder Mayer, Naturkundemuseum Berlin
91130	MMU82	IND4	KY052105	nasal bone	India	India		Dr. Frieder Mayer, Naturkundemuseum Berlin
70927	MMU83	SRI2	KY052117	nasal bone	Sri Lanka	Sri Lanka		Dr. Frieder Mayer, Naturkundemuseum Berlin
11959	MMU86	THA5	KY052106	nasal bone	Thailand	Thailand		Dr. Frieder Mayer, Naturkundemuseum Berlin
91133	MMU87	IND8	KY052107	nasal bone	India	India		Dr. Frieder Mayer, Naturkundemuseum Berlin
RMNH.MAM.33818	MMU90	JAV10	KY052152	nasal bone	West Java	Java	1930	Pepijn Kamminga, Naturalis Leiden
RMNH.MAM.36981	MMU91	THA3	KY052108	tissue from skull	North Thailand	Thailand	1989	Pepijn Kamminga, Naturalis Leiden
RMNH.MAM.33814	MMU95	LSI3	KY052153	tissue from skull	Bangka Island	Banka		Pepijn Kamminga, Naturalis Leiden
RMNH.MAM.4929	MMU100	JAV12	KY052154	nasal bone	Java	Java		Pepijn Kamminga, Naturalis Leiden
RMNH.MAM.4916	MMU102	IND1	KY052109	nasal bone	North India	India		Pepijn Kamminga, Naturalis Leiden
RMNH.MAM.29758.a	MMU105	BOR3	KY052155	tissue from skull	Central Borneo	Borneo	1894	Pepijn Kamminga, Naturalis Leiden
RMNH.MAM.29754.a	MMU107	BOR1	KY052156	tissue from skull	Borneo	Borneo		Pepijn Kamminga, Naturalis Leiden
74035/009957	C.2	CHI2	KY052110	nasal bone	Yunnan China	China		Douglas Yu, The Ecology, Conservation, and Environment Center
RJT118	RJT118	LAO1	KY052082		Laos	Laos		R. J. Timmins
M2.3	M2.3	VIE3	KY052083	tissue from skull	North C. Vietnam	North Vietnam	2011	Dr. Minh Le, Center for Natural Resources & Environmental Studies
M2.9	M2.9	VIE7	KY052084	bone	North C. Vietnam	North Vietnam	2010	Dr. Minh Le, Center for Natural Resources & Environmental Studies
M2.14	M2.14	VIE2	KY052085	bone	Central Vietnam	Central Vietnam	2011	Dr. Minh Le, Center for Natural Resources & Environmental Studies

M3.8	M3.8	VIE11	KY052086	bone	North E. Vietnam	North Vietnam		Dr. Minh Le, Center for Natural Resources & Environmental Studies
M5.11	M5.11	VIE13	KY052087	dry skin	North Vietnam	North Vietnam		Dr. Minh Le, Center for Natural Resources & Environmental Studies
M6.5	M6.5	VIE8	KY052088	bone	North C. Vietnam	North Vietnam		Dr. Minh Le, Center for Natural Resources & Environmental Studies
M6.12	M6.12	VIE1	KY052089	bone	Central Vietnam	Central Vietnam		Dr. Minh Le, Center for Natural Resources & Environmental Studies
M6.17	M6.17	VIE4	KY052090	bone	South Vietnam	South Vietnam	2012	Dr. Minh Le, Center for Natural Resources & Environmental Studies
x15	x15	VIE9	KY052111	bone	North C. Vietnam	North Vietnam		Dr. Minh Le, Center for Natural Resources & Environmental Studies
x17	x17	VIE12	KY052112	bone	North C. Vietnam	North Vietnam		Dr. Minh Le, Center for Natural Resources & Environmental Studies
x19	x19	VIE10	KY052113	bone	North C. Vietnam	North Vietnam		Dr. Minh Le, Center for Natural Resources & Environmental Studies
x20	x20	VIE6	KY052114	bone	North C. Vietnam	North Vietnam		Dr. Minh Le, Center for Natural Resources & Environmental Studies
x39	x39	VIE5	KY052115	bone	North C. Vietnam	North Vietnam		Dr. Minh Le, Center for Natural Resources & Environmental Studies
mm13	mm13	MAL1	KY052118		Peninsular Malaysia	Peninsular Malaysia		Monash University, Malaysia
mm20	mm20	MAL2	KY052119		Peninsular Malaysia	Peninsular Malaysia		Monash University, Malaysia

Table S3. Long-range PCR primer sequences and annealing temperatures used for bait development.

	Position on ref.	Sequence 5'-3'	Annealing Temp.[a]
Fragment 1	2478<br>6926	F: CGATTAAAGTCCTACGTGATCTGAG<br>R: GTTATGATGTTGGCTTGAAACCAG	58 ^{o}C
Fragment 2	6506<br>12903	F: GCTATYATRGGAGGATTTGTTCAC<br>R: GATTAGGGCTGTTGTRGTAAATG	58 ^{o}C
Fragment 3	12339<br>2548	F: TTACAAATCTTAACGCCTGAGACTTC<br>R: TAGATAGAAACCGACCTGGATTACTC	58 ^{o}C

[a] PCR was done with MyFi™ Mix (Bioline GmbH, Germany) with 1x MyFi Mix, 0.4 µM each primer and water to the final volume of 50 µL for each of the fragments separately, in a total of 35 amplification cycles.

Table S4. Sequencing results from all samples used in this study, with indication of sequencing platform, percentage of reads on target, average base coverage depth and percentage of reference genome covered with at least 3x coverage.

Sample Name	Captured	Sequencing platform	Reads on target	Average read depth	% genome covered ≥ 3x
BAL1	yes	PGM	53.65%	109.24	100
JAV13	yes	PGM	45.29%	294.49	100
THA2	yes	PGM	20.92%	45.73	99.9
IND5	no	Illumina	0.54%	90.76	100
IND2	yes	PGM	18.15%	16.17	99.4
CHI3	no	Illumina	0.06%	11.13	92.4
IND10	yes	PGM	46.65%	257.54	100
BOR2	yes	Illumina	2.21%	14.07	93.7
LOM2	yes	Illumina	36.22%	124.63	100
SRI1	yes	PGM	20.46%	112.53	99.2
CHI1	yes	PGM	4.30%	186.14	100
LSI1	yes	Illumina	45.26%	172.8	100
BOR7	yes	PGM	10.17%	39.69	99.9
SUM6	yes	PGM	36.05%	96.74	100
SUM1	yes	PGM	47.57%	64.02	99.8
JAV17	yes	PGM	71.62%	65.65	99.8
JAV15	yes	PGM	65.44%	88.36	100
JAV5	yes	PGM	55.88%	163.68	100
BOR5	yes	PGM	6.24%	6.87	88.1
BOR6	yes	PGM	31.17%	7.97	93.9
BOR4	yes	PGM	18.20%	27.78	99.9
THA6	yes	PGM	26.33%	178.63	100
JAV6	yes	PGM	6.35%	187.35	100
JAV11	yes	PGM	40.60%	36.44	99
SUM4	yes	PGM	44.44%	278.49	100
SUM2	yes	PGM	61.77%	27.59	99
JAV14	yes	PGM	30.97%	144.77	100
JAV1	no	Illumina	0.92%	74.02	100
JAV16	yes	PGM	30.47%	36.33	99.5
JAV3	no	Illumina	0.18%	14.49	92.5
JAV8	yes	Illumina	4.88%	25.39	96.1
SUM5	no	Illumina	0.05%	100.91	100
JAV7	yes	Illumina	3.88%	79.14	98.2
JAV2	yes	Illumina	9.26%	89	99.6
JAV4	yes	PGM	50.03%	10.66	89.7
SUM3	yes	PGM	24.66%	72.7	100
LOM1	yes	PGM	28.73%	37.86	99.1
LOM3	yes	PGM	33.09%	35.93	99.3
IND6	yes	PGM	50.73%	47.47	99.9
JAV9	yes	PGM	27.96%	20.03	98.4
NEP1	yes	PGM	59.64%	31.76	100
LSI2	yes	PGM	16.87%	17.46	98.8
IND7	yes	PGM	47.52%	146.08	100
THA4	no	Illumina	0.03%	8.43	86.1
THA1	yes	Illumina	14.59%	92.01	100
IND9	yes	PGM	33.76%	7.29	92.6
IND3	yes	Illumina	1.76%	22.85	98.17
IND4	yes	Illumina	8.41%	56.76	99.65
SRI2	yes	PGM	61.03%	51.33	98.6
THA5	yes	Illumina	29.52%	155.25	100

IND8	yes	Illumina	64%	218.42	100
JAV10	no	Illumina	0.07%	78.4	99.7
THA3	yes	PGM	39.65%	17.13	99.8
LSI3	yes	Illumina	18.50%	147.87	100
JAV12	yes	Illumina	3.52%	34.46	99.4
IND1	yes	PGM	74.84%	108.69	100
BOR3	yes	Illumina	19.56%	88.28	97.9
BOR1	yes	Illumina	39.98%	115.74	99.3
CHI2	no	Illumina	0.19%	49.41	100
LAO1	no	Illumina	0.18%	53.8	99.05
VIE3	no	Illumina	0.04%	12.66	96.7
VIE7	no	Illumina	0.03%	11.42	95.4
VIE2	no	Illumina	0.07%	16.19	92.5
VIE11	no	Illumina	0.06%	21.06	97
VIE13	no	Illumina	0.08%	19.42	99.7
VIE8	no	Illumina	0.10%	29.03	96.8
VIE1	no	Illumina	0.03%	8.19	90.6
VIE4	no	Illumina	0.03%	11.25	94.1
VIE9	no	Illumina	0.12%	37.95	99.9
VIE12	no	Illumina	0.20%	70.42	100
VIE10	no	Illumina	0.07%	31.10	100
VIE6	no	Illumina	0.03%	9.38	94.2
VIE5	no	Illumina	0.06%	30.30	99.9
MAL1	no	Illumina	0.93%	66.63	100
MAL2	no	Illumina	0.58%	57.01	100

Table S5: Number of Variable sites (V) and Parsimony informative sites (Pi) per coding gene, throughout the full mitogenomes obtained.

	ND1	ND2	Cox1	Cox2	Cox3	ND3	ND4L	ND4	ND5	ND6	Cytb	D-loop
**V**	126	147	163	76	108	43	38	170	277	64	182	157
**Pi**	102	101	111	54	79	31	27	132	191	45	127	120

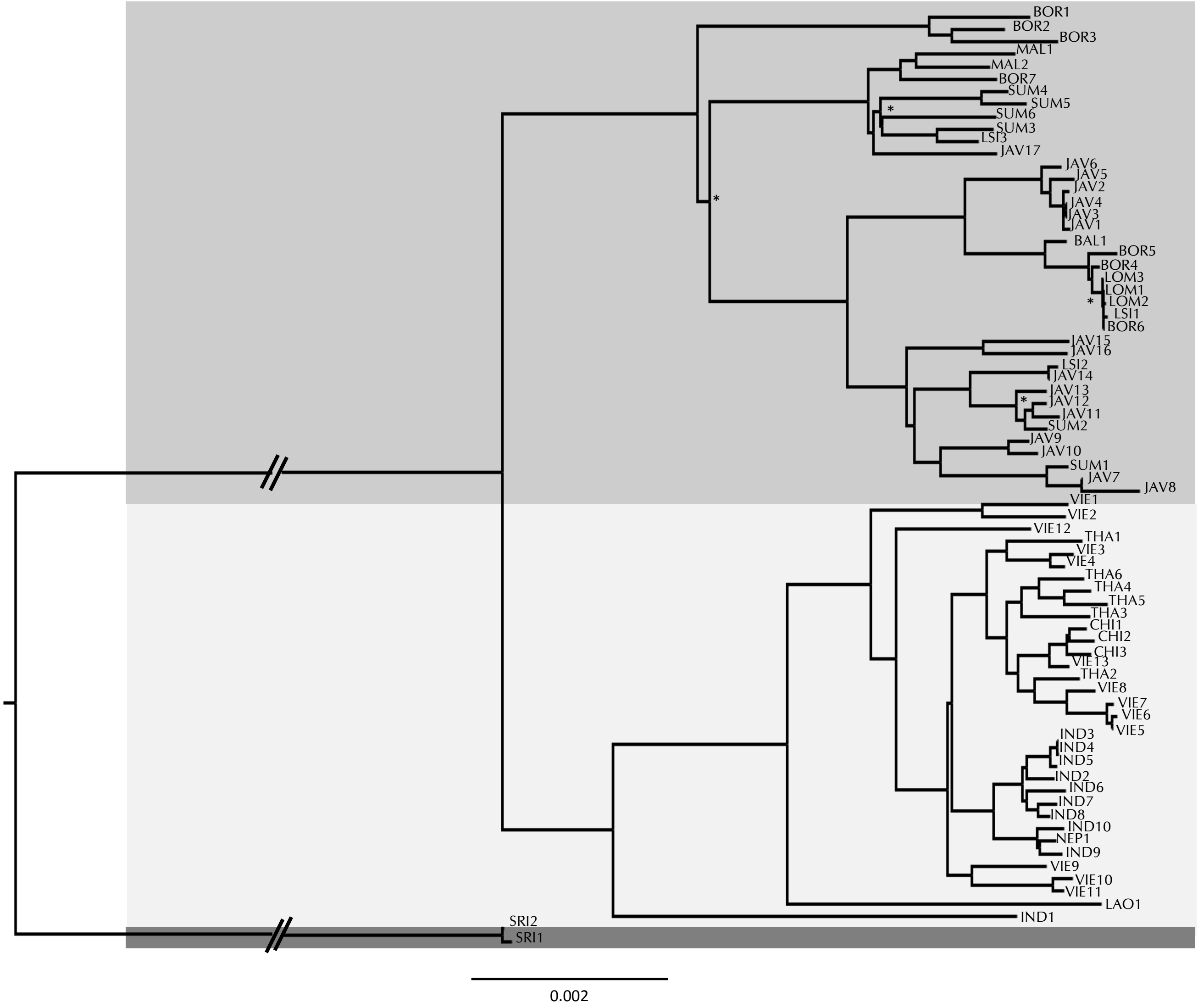

Figure S1. Best tree obtained with Maximum Likelihood analyses with RAxML. The same topology is recovered as with Bayesian inferences, with three distinct mitochondrial clades. All branches are supported with at least 90% bootstrap support, except when indicated otherwise with *.

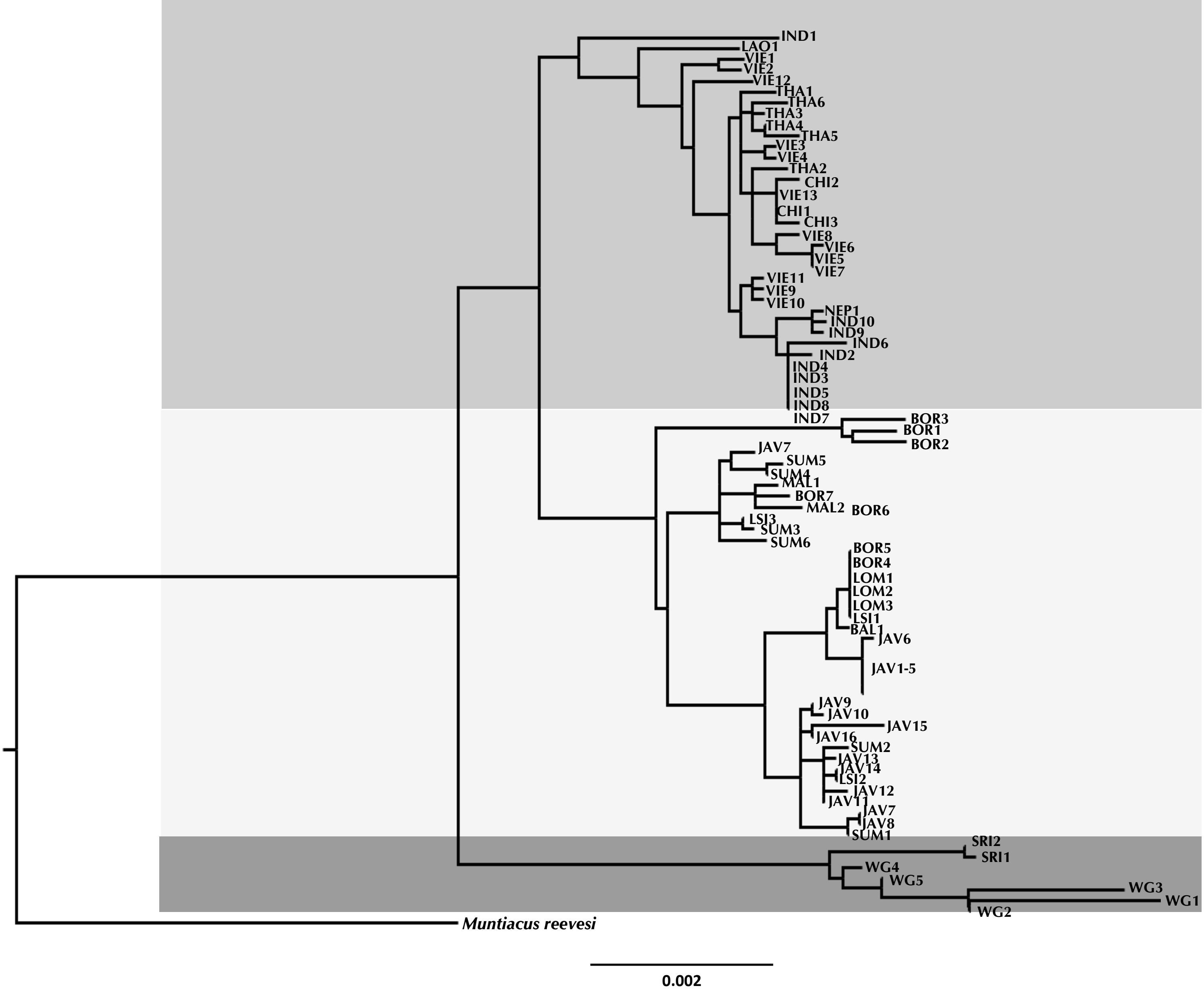

Figure S2. Best tree obtained with Maximum Likelihood analyses of the Cytochrome B gene of all red muntjac samples included in this study and five additional Genbank sequences from the Western Ghats. This tree was obtained with RAxML and shows similar topology as the trees with full mitogenome, although bootstrap values are generally lower. This result shows the relationship between Sri Lanka and Western Ghats populations, as they cluster together in the same clade.
